# Segregation and linkage analysis for longitudinal measurements of a quantitative trait

**DOI:** 10.1186/1471-2156-4-S1-S21

**Published:** 2003-12-31

**Authors:** Conway Gee, John L Morrison, Duncan C Thomas, W James Gauderman

**Affiliations:** 1 Department of Preventive Medicine, University of Southern California, 1540 Alcazar Street, Los Angeles, California, USA

## Abstract

We present a method for using slopes and intercepts from a linear regression of a quantitative trait as outcomes in segregation and linkage analyses. We apply the method to the analysis of longitudinal systolic blood pressure (SBP) data from the Framingham Heart Study. A first-stage linear model was fit to each subject's SBP measurements to estimate both their slope over time and an intercept, the latter scaled to represent the mean SBP at the average observed age (53.7 years). The subject-specific intercepts and slopes were then analyzed using segregation and linkage analysis. We describe a method for using the standard errors of the first-stage intercepts and slopes as weights in the genetic analyses. For the intercepts, we found significant evidence of a Mendelian gene in segregation analysis and suggestive linkage results (with LOD scores ≥ 1.5) for specific markers on chromosomes 1, 3, 5, 9, 10, and 17. For the slopes, however, the data did not support a Mendelian model, and thus no formal linkage analyses were conducted.

## Introduction

In the conventional epidemiology literature, much has been written about utilizing data in which measurement of quantitative traits are periodically taken from each subject over time [1-3]. However, relatively little has been written regarding the use of longitudinal data in genetic epidemiological studies. One method proposed by Levy et al. [4] utilized the within-subject mean SBP to summarize the longitudinal measurements for each subject. In Levy's analysis of data from the Framingham Heart Study, a sample-wide regression was used to model SBP over time as a linear function of age and body mass index after adjusting for sex, cohort group, and hypertension treatment in a first-stage analysis. Residuals for each subject from the regression analysis, averaged over time, were then used as continuous phenotype data in a linkage analysis in the second stage of the analysis.

In this paper, we also adopt a two-stage modeling approach. In our first stage, we fit a linear regression of SBP on age to obtain subject-specific intercepts and slopes. This first-stage model includes adjustment for any time-varying covariates of interest, such as calendar year, body mass index, and hypertension treatment. Also estimated in this first stage are the subject-specific standard errors of the corresponding intercepts and slopes. The second stage analysis consists of a segregation analysis of the subject-specific intercepts from the first stage model, and a separate segregation analysis of the slopes. We claim that the standard errors from the first stage should be used in weighting the contribution of each subject in the segregation analysis, and we describe how this can be accomplished. Based on the results of the segregation analyses, we conducted a genome screen using parametric linkage analysis applied to all pedigrees in the Framingham Heart Study. We demonstrate increased LOD scores using the weighed analysis, compared with the analogous approach that does not use weights.

## Methods

### General approach

Let Y_*ij *_denote the SBP of the i^th ^subject at the j^th ^study visit, *T*_*ij *_be the corresponding age of the subject at the visit, and let ***X***_*ij *_denote a matrix of time-dependent covariates. We propose using a first-stage model of the form

*Y*_*ij *_= *a*_*i *_+ *b*_*i *_(*T*_*ij *_- ) + **γ'*X***_*ij *_+ *e*_*ij*_,     (1)

where  is the overall mean age in the sample and *e*_*ij*_ are residuals, assumed to be independent and normally distributed with mean 0 and variance ω^2^. The goal of this first-stage model is to estimate the subject-specific intercepts (*a*_*i*_) and slopes on age (*b*_*i*_), and their corresponding standard errors. We denote these standard errors by s_*ai *_for intercepts and s_*bi *_for slopes. Note that the intercepts have the interpretation as the predicted mean *Y *at age  when all *X *values are zero. We center any continuous *X *variables on their corresponding sample means to increase interpretability of the intercepts.

The second-stage model utilizes the first-stage intercepts *a*_*i *_and slopes *b*_*i *_as continuous phenotype data in a genetic analysis. We first perform segregation analysis to determine the evidence for a Mendelian gene and to estimate the associated model parameters. For analysis of the intercepts, the penetrance model used in the segregation analysis has the form

*a*_*i *_= α + β*G*_*i *_+ **η'*X*_*i *_**+ *e*_*i*_,     (2)

where *G*_*i *_is a covariate based on an unobserved major gene *g*_*i*_, and ***X***_*i *_is a matrix of time-independent covariates. An analogous model was used for the slopes. The residual *e*_*i *_is assumed to be normally distributed with mean 0 and variance (σ^2 ^+ s_*ai*_^2^), where s_*ai*_^2 ^is the square of the first-stage standard error of the intercept, and σ^2 ^is the between-subject residual variance to be estimated. Note that this variance expression has the effect of weighting each subject's contribution to the genetic analysis based on the precision (standard error) of their intercept estimate. We thus denote the use of this variance for *e*_*i *_as a 'weighted' analysis. Generally speaking, these first-stage standard errors will be smallest for those with many measurements, and with measurements at ages that span the overall average age . One could also perform an 'unweighted' analysis by assuming that the variance of *e*_*i *_was simply σ^2^, which would treat the intercepts for all subjects as equally informative.

To estimate the parameters of the above model, we maximized the likelihood



where the *F *indexes family, ***g***_F _is a vector of unobserved major genotypes, and ***Y***_F _and ***X***_F _are the trait and covariate data for family F. The parameters Ω = {α, β, η, σ } are the parameters of the penetrance model, *q*_A _is the population frequency of the variant allele 'A', and τ = {τ_AA_, τ_Aa_, τ_aa_} are the probabilities that a parent with the subscripted genotype transmits an 'A' to their offspring. Computation of the above likelihood requires use of the peeling algorithm [5,6]. We considered six models in the segregation analysis: four Mendelian models (dominant, recessive, additive, and codominant), a no-major-gene model that included only measured covariates, and a general transmission model. In the general transmission model, τ_AA_, τ_Aa_, and τ_aa _were treated as free parameters to be estimated. This general model was compared to the Mendelian models, in which τ_AA_, τ_Aa_, and τ_aa _were constrained to their theoretical values of 1.0, 0.5, and 0.0, respectively. Likelihood ratio tests (LRTs) were used to compare the general model to the Mendelian models, and also to the no-major-gene model. We also computed Akaike's Information Criteria (AIC) for each model as -2(log-likelihood at the maximum likelihood estimator (MLE)) + 2(number of model parameters estimated). A lower AIC indicates a more parsimonious model.

### Application to the Genetic Analysis Workshop 13 (GAW13) Framingham Heart Study data set

The GAW13 data set of the Framingham Heart Study included a total of 4692 subjects, of which 1213 subjects provided longitudinal observation data from the first cohort, and 1672 subjects from the offspring cohort. The outcome variable of interest in this paper was systolic blood pressure (SBP). A natural log transform was used to linearize the SBP relationship with age; thus *Y*_*ij *_in equation (1) is ln(SBP_*ij*_). Only observations with age in the range 30 to 80 were utilized, to further linearize the relationship between ln(SBP) and age. The average age was  = 53.7. Time-dependent covariates defining ***X***_*ij *_in equation (1) included body mass index (BMI), calendar year (CY), CY^2^, hypertension treatment (HRX), CY × HRX, CY × male, CY × cohort, CY × BMI, CY × age, male × age, and BMI × HRX. The continuous variables BMI and CY were centered on their respective sample means, while HRX and male were indicators of treatment status and male sex, respectively. The CY^2 ^term was included to account for observed nonlinearity between SBP and CY. The intercepts from the first-stage model have interpretation as the subject-specific mean ln(SBP) adjusted to a female, untreated person of average age (53.7 years) and BMI (26.3 kg/m^2^) in calendar year 1969.5. PROC MIXED in SAS, Release 8.2 (SAS Institute, Cary NC), was used to fit the first-stage model and obtain person-specific intercepts and slopes, and their respective standard errors.

A total of 2883 person-specific intercepts (*a*_*i *_values) and 2787 person-specific slopes (*b*_*i *_values) were obtained from the first-stage analysis. These estimates were used as trait data in the second-stage segregation and linkage analyses. Covariates ***X***_*i *_in equation (2) included male sex and cohort, the latter an indicator of membership in Cohort 2. We fit the segregation and linkage models using a version of the Genetic Analysis Package (GAP, Epicenter Software, Pasadena, CA), modified by one of the authors (WJG) to utilize s_*ai*_^2 ^(and s_*bi*_^2^) in a weighted analysis. As will be demonstrated below, a Mendelian model was supported for the intercepts, but not for the slopes. We therefore focused our linkage analysis only on the intercepts. We fixed the segregation-model parameters to their MLEs from the weighted analysis, and performed two-point LOD-score linkage analysis to estimate the recombination fraction (θ) between *g *and each of 399 markers. Allele frequencies at each marker locus were fixed to the values provided with the data. For comparison, we also performed an unweighted linkage analysis, in which a segregation analysis was re-run without standard error weights, and these MLEs then used in linkage analysis.

## Results

### Segregation analysis

Segregation analysis of the intercepts supported a Mendelian codominant model, with strong evidence of a genetic effect (Table 1). Specifically, compared to the general model, the Mendelian codominant model did not fit significantly worse (χ^2 ^= 1.7, *p *= 0.43, conservatively assuming two degrees of freedom). On the other hand, the remaining Mendelian models and the no-major-gene model could be rejected (*p *< 0.001 for each). The codominant model also provided the lowest AIC, again indicating that this model provided the best fit to the data. The estimated allele frequency from this model was *q*_A _= 0.31, translating into 48% of subjects with *g *= aa, 42% with *g *= Aa, and 10% with *g *= AA. Compared with subjects with *g *= aa, SBP was estimated to be 12% higher (exp(0.115)) for subjects with *g *= Aa, and 33% higher (exp (0.283)) for subjects with *g *= AA. Segregation analysis of the slopes, on the other hand, did not support evidence of Mendelian transmission (Table 2). Specifically, the general model fit significantly better than any of the Mendelian models (*p *< 0.001), and provided the lowest AIC. The estimate of the transmission parameter τ_AA _was 0.0, far from its Mendelian expectation of 1.0.

### Linkage analysis

**Figure 1:**
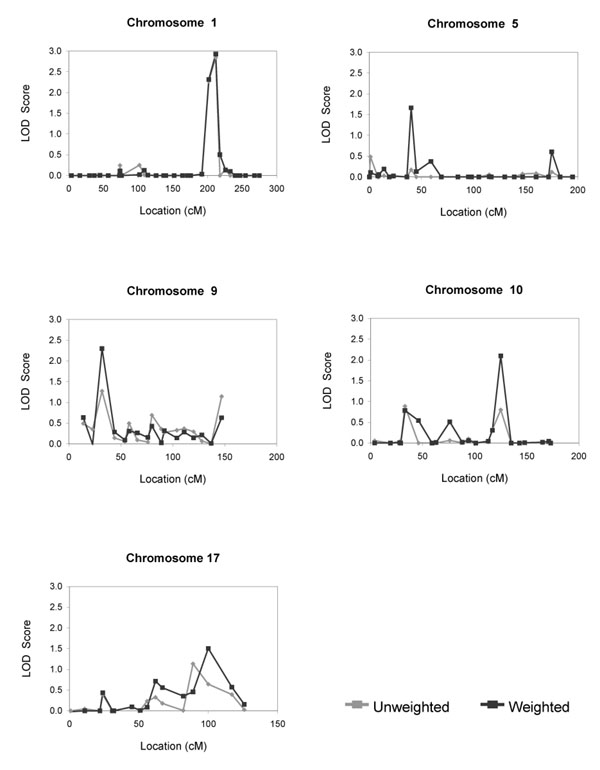
LOD scores on four chromosomes based on genetic analyses of intercepts that either do (weighted) or do not (unweighted) incorporate first-stage standard errors

Given the findings in segregation analysis, linkage analysis was conducted only on the intercepts. The parameters of the segregation model were fixed to the values shown for the Mendelian codominant model in Table 1. Two-point linkage analysis was conducted, i.e., markers were considered one at a time in separate analyses. Each analysis utilized standard-error based weights as in the segregation analyses. For comparison, we also repeated the segregation and linkage analyses using unweighted analysis, i.e., not using first-stage standard errors as weights. Table 3 shows the markers that yielded a LOD score of at least 1.5 using either the weighted or unweighted approach. The strongest evidence of linkage was found at marker positions 202 (LOD = 2.3) and 212 (LOD = 2.9) on chromosome 1, position 32 (LOD = 2.3) on chromosome 9, and position 125 (LOD = 2.1) on chromosome 10 in the weighted analysis, and at position 153 (LOD = 2.0) on chromosome 3 in the unweighted analysis. Weaker evidence for linkage was observed on chromosomes 5 and 17 in the weighted analysis. The weighted analysis generally led to larger LOD scores than the unweighted analysis. To further compare the weighted and unweighted approaches, Figure 1 shows plots of the LOD scores at all markers on chromosomes 1, 5, 9, 10, and 17. While the LOD scores for the two approaches have similar trends across markers, the weighted analysis provides more striking peaks, particularly on chromosomes 9, 10, and 17.

## Discussion

Our segregation analysis indicates that SBP, specifically average SBP at age 53, has a significant genetic basis. We estimated that approximately half of the population carries a genotype (*g *= Aa or AA) that leads to some elevation in average SBP relative to genetically normal (*g *= aa) individuals. Subsequent analysis revealed modest evidence of linkage on chromosomes 1 (202 and 212 cM), 5 (40 cM), 9 (32 cM), 10 (125 cM), and 17 (100 cM). Levy et al. [4] also found evidence of linkage at the same position on chromosome 10 (125 cM), but at different positions on chromosomes 5 (59 cM) and 10 (125 cM). Rao et al. [7] also reported linkage to position 125 cM on chromosome 10, while Briollais et al. [8] reported evidence of linkage at position 212 cM on chromosome 1.

The appropriate use of standard errors from the first stage model in the second stage model generally resulted in larger LOD scores than were obtained in an unweighted analysis. However, since our application was to a real data set for which we do not know the truth, we cannot conclude with certainty that the use of weights will generally lead to more significant linkage peaks. Our proposed two-stage approach should be evaluated further using simulated data.

We did not find support for an effect of a Mendelian gene on SBP slope in our segregation analysis. This may be a consequence of the model form we applied in our analysis, for example in our assumption that any genetic effect was mediated through a single major gene. If multiple genes affect SBP change over time, our model may have had low power to detect a genetic signal. As a 'fishing expedition', we ignored our lack of support for a Mendelian model and performed a genome screen for linkage to SBP slopes. The segregation model parameters were fixed to the values for the Mendelian codominant model shown in Table 2. This analysis revealed no LOD scores that exceeded 1.5 at any marker. This failure to find any linkage signals may again be a consequence of poor power, or it may reflect our segregation-analysis finding that slopes are not determined by a Mendelian gene.

Although we developed a two-stage modeling approach in this paper, we believe that it would be preferable to combine the first and second stage models into a single analysis. This would consist of performing a joint segregation and linkage analysis of the original, repeated SBP measurements on each subject. Some advantages of this approach are that parameter estimates in each model would be mutually adjusted for one another, and subjects with more observations would naturally contribute more information to parameter estimation and testing. To achieve this latter quality in a two-stage analysis required the incorporation of standard-error derived weights from the first-stage model into the second-stage model, as described in this paper. The primary deterrent to implementing a joint approach lies in the computational difficulty of simultaneously fitting a longitudinal model and summing over a large space of unobserved genotypes. One could consider using Markov chain Monte Carlo methods to solve this computational difficulty (see Palmer et al. [9] and Scurrah et al. [10]), and we would encourage formal comparison of these approaches to a two-stage approach to assess their relative merits.

There are some difficult issues in this particular data set that we have not addressed. First is the issue of how to best handle hypertension treatment (HRX). We chose to include HRX as a time-dependent covariate in our first-stage model. However, since the decision to treat is based on SBP, this approach may lead to invalid estimates of the HRX effect, and may ultimately affect our genetic inferences as well. Levy et al. [4] propose a different approach for dealing with HRX in the analysis of longitudinal SBP. Clearly, more work is required to better understand how to best adjust for covariates that are themselves determined by the outcome variable. Another important issue is the problem of missing data. In our analysis, we used only observations at each time point that had complete outcome and covariate data. The elimination of missing observations may introduce bias if the missingness is related to the condition of the subject at that time (see Kang et al. [11], for a longer discussion). Furthermore, if missingness patterns are correlated within families, results from segregation and linkage analyses may be further misrepresented.

We adopted a parametric modelling approach in our genetic analysis. An advantage of this approach is that it utilizes all available data in each pedigree. A disadvantage, however, is that the model form was likely misspecified, particularly if SBP is determined by several genes with differing allele frequencies and effects on the trait. As an alternative, one could replace our second-stage parametric model with a weighted nonparametric linkage approach, for example using a variance components (VC) [12] or Haseman-Elston (HE) [13,14] model. In a VC analysis of intercepts (or slopes), one could add a subject-specific component to the variance based on the first-stage standard-error. In the HE approach, one could regress some function of the first stage intercepts for a pair of relatives (e.g., the squared difference in intercepts between sib pairs) on the proportion of alleles shared identical by descent at a marker locus. The delta method can be utilized to calculate the variance of the squared sib-pair difference as a function of the first-stage, subject-specific standard errors. The inverse of the variances for each sib pair could then be used as weights in the HE regression. The performance of weighted VC and HE linkage analysis, relative to each other and to unweighted analysis, is a topic for future research.

In conclusion, we have proposed a two-stage modelling approach to the genetic analysis of longitudinal data for a quantitative trait. Additional work is necessary to evaluate the method, including simulation studies and comparisons to other two-stage and joint-analysis approaches.

## Figures and Tables

**Table 1:** Weighted segregation analysis of intercepts*

	Hypothesis											
			Mendelian									
Segregation Parameter	General		Codominant		Dominant		Recessive		Additive		No Major Gene	
	Estimate	SE	Estimate	SE	Estimate	SE	Estimate	SE	Estimate	SE	Estimate	SE
*Intercept*	4.769	0.0078	4.771	0.0072	4.802	0.0052	4.801	0.0051	4.776	0.0065	4.846	0.0041
β_*cohort*_	-0.088	0.0077	-0.092	0.0043	-0.092	0.0046	-0.091	0.0047	-0.092	0.0043	-0.085	0.0049
β_*Sex*_	0.011	0.0046	0.011	0.0046	0.012	0.0046	0.010	0.0047	0.012	0.0046	0.005	0.0049
β_*AA*_	0.288	0.0143	0.283	0.0142	0.165	0.0066	0.167	0.0072	0.269	0.0107	—	—
β_*Aa*_	0.118	0.0076	0.115	0.0078	0.165^A^	—	0.000^B^	—	0.135^C^	—	—	—
*q*_*A*_	0.323	0.0539	0.305	0.0373	0.139	0.0180	0.511	0.0304	0.257	0.0285	—	—
σ^2^	0.004	0.0004	0.004	0.0004	0.006	0.0004	0.006	0.0004	0.004	0.0004	0.011	0.0004
τ_*aa*_	0.000	0.0000	0.000^D^	—	0.000^D^	—	0.000^D^	—	0.000^D^	—	—	—
τ_*Aa*_	0.476	0.0610	0.500^D^	—	0.500^D^	—	0.500^D^	—	0.500^D^	—	—	—
τ_*AA*_	0.935	0.0611	1.000^D^	—	1.000^D^	—	1.000^D^	—	1.000^D^	—	—	—

-2(log-likelihood)	-3482.64		-3480.94		-3400.16		-3376.52		-3463.32		-3155.59	
*p*-value^E^	—		0.43		< 0.001		< 0.001		< 0.001		< 0.001	
AIC^F^	-3462.64		-3466.94		-3388.16		-3364.52		-3451.32		-3147.59	

*The outcome being modeled in equation (2) is *a*_*i *_from equation (1). ^A^Constrained to equal β_*AA*_. ^B^Constrained to equal 0. ^C ^Constrained to equal 1/2 β_*AA*_. ^D ^Parameter value is fixed. ^E^*p*-value based on a likelihood ratio test with the general model as the base model.^F^AIC = -2(log-likelihood) + 2(number of free parameters).

**Table 2:** Weighted segregation analysis of slopes*

	Hypothesis											
	
			Mendelian									
					
Segregation Parameter	General		Codominant		Dominant		Recessive		Additive		No Major Gene	
	Estimate	SE	Estimate	SE	Estimate	SE	Estimate	SE	Estimate	SE	Estimate	SE

*Intercept*	3.205	0.1814	3.500	0.1932	3.790	0.2246	4.139	0.1489	3.744	0.2081	4.265	0.1485
β_*cohort*_	-3.541	0.2393	-3.819	0.2094	-3.785	0.2092	-3.788	0.2143	-3.793	0.2090	-3.726	0.2113
β_*Sex*_	-1.621	0.1981	-1.623	0.1965	-1.584	0.2001	-1.682	0.1907	-1.580	0.1897	-1.620	0.1936
β_*AA*_	16.614	1.7795	16.625	2.4421	6.742	1.2112	14.296	2.1622	12.821	2.1109	—	—
β_*Aa*_	4.443	0.5003	3.525	0.8312	6.742^A^	—	0.000^B^	—	6.411^C^	—	—	—
*q*_*A*_	0.199	0.0584	0.110	0.0265	0.042	0.0195	0.130	0.0269	0.047	0.0188	—	—
σ^2^	0.485	0.1782	1.849	0.5964	2.384	0.7088	3.384	0.5886	2.206	0.5857	4.949	0.6492
τ_*aa*_	0.000	0.0000	0.000^D^	—	0.000^D^	—	0.000^D^	—	0.000^D^	—	—	—
τ_*Aa*_	0.390	0.0694	0.500^D^	—	0.500^D^	—	0.500^D^	—	0.500^D^	—	—	—
τ_*AA*_	0.000	0.0000	1.000^D^	—	1.000^D^	—	1.000^D^	—	1.000^D^	—	—	—

-2(log-likelihood)	17811.58		17824.66		17839.45		17828.49		17837.35		17867.83	
*p*-value^E^	—		< 0.001		< 0.001		< 0.001		< 0.001		< 0.001	
AIC^F^	17831.58		17838.66		17851.45		17840.49		17849.35		17875.83	

*The outcome being modeled in equation (2) is 1000 × *b*_*i*_, the subject-specific slope from equation (1). ^A^Constrained to equal β_*AA*_. ^B^Constrained to equal 0. ^C^Constrained to equal 1/2 β_*AA*_. ^D^Parameter value is fixed. ^E^*p*-value based on a likelihood ratio test with the general model as the base model.^F^AIC = -2(log-likelihood) + 2(number of free parameters).

**Table 3:** Markers with LOD score ≥ 1.5 based on two-point linkage analysis* of subject-specific SBP intercepts

			Weighted		Unweighted	
				
Chromosome	Location (cM)	Marker	LOD	*p*-value	LOD	*p*-value
1	202	GATA7C01	2.31	0.0005	2.27	0.0006
1	212	GATA48B01	2.93	0.0001	2.84	0.0002
3	153	GATA4A10	0.83	0.026	2.00	0.0012
5	40	GATA145D09	1.66	0.0028	0.17	0.19
9	32	GATA27A11	2.30	0.0006	1.27	0.0078
10	125	GATA64A09	2.10	0.0009	0.80	0.028
17	100	GATA28D11	1.50	0.0042	0.64	0.0428

* Assuming the Mendelian codominant model (see Table 1)
